# Exploring the Association Between Internet Use and Dietary Habits of Adolescents and University Students in Greece: A Pilot Study

**DOI:** 10.3390/nu18071085

**Published:** 2026-03-28

**Authors:** Christina Stavraki, Nikolaos Georgiadis, Eleni Kornarou, Artemis K. Tsitsika, Theodoros N. Sergentanis, Tonia Vassilakou

**Affiliations:** 1 Department of Public Health Policy, University of West Attica, 11521 Athens, Greece; tinastavraki@gmail.com (C.S.); nikgeorgiadis@uniwa.gr (N.G.); ekornarou@uniwa.gr (E.K.); tsergentanis@uniwa.gr (T.N.S.); 2 Adolescent Health Unit, 2nd Department of Pediatrics, “P. & A. Kyriakou” Children’s Hospital, Medical School, National and Kapodistrian University of Athens, 11527 Athens, Greece; info@youth-health.gr

**Keywords:** internet addiction (IA), disordered eating behavior (DEB), mediterranean diet (MD) compliance, high school students, university students

## Abstract

**Background/Objectives:** Adolescents and university students appear to be at increased risk for internet addiction (IA), while disordered eating behavior (DEB) is common in these age groups. At the same time, adherence to the Mediterranean diet (MD) has declined in many countries. This study aimed to explore the potential association between IA, DEB and MD compliance among high school and university students. **Methods:** A total of 212 students aged 15–24 years participated in this cross-sectional study conducted in Greece. Data were collected via an online questionnaire including the Internet Addiction Test (IAT), KIDMED, and EAT-26 scales. Descriptive statistics, chi-square tests, and univariate and multivariate logistic regression analyses were performed. **Results:** Most participants demonstrated normal internet use (69.8%), while 23.1% showed mild IA and 7.1% moderate IA. Regarding dietary habits, 9.4% had low MD adherence, 52.8% moderate and 37.7% high adherence. A total of 15.6% scored above the EAT-26 cut-off, indicating risk for disordered eating behavior. IA was only significantly associated with urbanization (*p* = 0.014). MD adherence was not associated with gender, urbanization, financial or education status. Multivariate logistic regression showed that female gender (OR = 9.28, 95% CI: 2.10–40.91, *p* = 0.003) and moderate IA (OR = 6.70, 95% CI: 1.71–26.35, *p* = 0.006) were significant predictors of disordered eating, while educational status and MD adherence were not significant predictors. **Conclusions:** Moderate IA and female gender were strongly associated with an increased risk for disordered eating. Further qualitative and clinical studies are needed to better understand the interaction between IA, eating behaviors, and dietary patterns in young people.

## 1. Introduction

Adolescence is a period of rapid physical, mental and social growth that bridges childhood to adulthood [1,2]. According to the Lancet Commission on Adolescent Health and Wellbeing, young people include younger adolescents (10–14 years), older adolescents (15–19 years), and young adults (20–24 years) [1]. A balanced diet sets the foundations for the adolescent’s health and well-being as an adult [3]. The development of body image is necessary for the development of the individual’s identity [4].

Considering the increasing amount of time that adolescents spend with their peers outside the family home and the school environment, more opportunities for unhealthy food choices are created [5]. Although a balanced diet in adolescence is of high importance, adolescents often consume high amounts of sugary or processed foods and low amounts of fruits and vegetables [6,7]. In addition, the socio-economic status of the family seems to play a prominent role in shaping the eating habits of adolescents; according to a recent systematic review, unhealthy food consumption was associated with a low socioeconomic status [8].

Young adulthood marks a transitional period between adolescence and adulthood. However, this period has been associated with negative changes in young people’s eating habits, as many young adults live away from their family homes, taking on the preparation of their daily meals, while they lack food shopping, meal preparation and meal planning experience. Most students tend to consume ready-made snacks, fast food, sweets and soft drinks, while the consumption of fruits and vegetables is clearly limited [9,10]. Young students are among the biggest consumers of sugary drinks and fast food and report low fruit and vegetable consumption [11]. The early years of university life have been associated with weight gain and higher prevalence of overweight and obesity among students [12].

The Mediterranean diet (MD) has been recognized as a dietary pattern that has multiple benefits in people’s health [13,14,15,16,17]; however, a large-scale survey in 41 countries, showed that the majority of countries appear to be moving away from the dietary pattern of MD, with Greece having the largest decrease among all Mediterranean countries [18].

Eating disorders (ED) including anorexia nervosa (AN), bulimia nervosa (BN) and Binge Eating Disorder (BED) are also frequent among adolescents and young adults [19,20].

Loss of control over internet use can create major issues relating to individual well-being, family relationships, social interactions, academic performance, and daily life functions, termed ‘internet addiction” (IA) [21]. IA has been considered as a form of behavioral addiction in association with decreased self-control and increased impulsivity [22,23]. Although IA can occur at almost any age, adolescents and students have been reported to be at greater risk [24,25,26]. IA has been associated with mood disorders [26], obesity and lack of physical activity [27,28], mental health conditions [27,29], sleep problems [30], and eye disturbances [31], as well as with EDs.

Since 2009, there has been growing research interest in the association of IA with eating habits, mainly of adolescents and young adults, as these are the age groups that are at greater risk for both IA and EDs [21,22,32,33,34,35]. IA has been associated with a negative impact on adolescents’ and young adults’ diet [24,36,37], as well as with EDs [21,22,38,39,40]. A meta-analysis conducted by Hinojo-Lucena et al. argued that adolescents and students with IA showed higher rates of EDs [22].

Research supporting the correlation between IA and EDs among adolescents and university students has been conducted in various countries, including Poland [21], France [34], Turkey [37,41], Egypt [33,36], China [35,42] and Bangladesh [43]; on the other hand, other studies suggested that further investigation is required in order to verify a statistically significant correlation between IA and EDs [24,33,40,41]. In Greece, to the best of our knowledge, there is no study exploring the possible correlation between IA, EDs and eating habits for the age groups of adolescents and young adult university students. Although some Greek studies have examined internet use or eating-related behaviors separately, research investigating the combined relationship between internet addiction, disordered eating behaviors and adherence to the Mediterranean diet in these populations remains limited. In light of the above, the purpose of this study was to explore whether IA is associated with EDs and adherence to MD among Greek high school adolescents (15–18 years old) and university students (18–24 years old).

## 2. Materials and Methods

### 2.1. Participants’ Recruitment

Participants were recruited through an anonymous online questionnaire created via Google Forms. The questionnaire was distributed via an electronic link through the internet and social media student groups, using a convenience sampling approach. Participation was voluntary, and individuals who met the inclusion criteria were invited to take part. Additionally, participants were encouraged to share the survey with other eligible individuals, resulting in elements of snowball sampling. The electronic format ensured the protection of participants’ personal data. Inclusion criteria were being a high school student (15–18 years) or a university student (18–24 years), ability to understand the Greek language, and provision of informed consent. Participants who did not meet these criteria or did not complete the questionnaire were excluded from the study. The participants completed the self-report questionnaire in the period of April–May 2022. Two hundred and twelve people (*N* = 212) participated in the survey.

### 2.2. Ethical Permission, Consent and Anonymity

Approval for the current study was granted by the Research Ethics and Deontology Committee of the University of West Attica (Registration number 20273/01-03-2022). The students received written information regarding their anonymity, their voluntary participation, the protection of their personal data, and the possibility of withdrawing at any time. Only the students that signed the relevant consent form were allowed to continue further and participate in the study. Students’ personal data were coded in order to ensure the protection of the provided information.

### 2.3. Questionnaire

The questionnaire included socio-demographic, anthropometric data and three validated scales for the Greek population, namely the Internet Addiction Test (IAT) [44,45], the Mediterranean Diet Quality Index for children and adolescents (KIDMED) [46,47] and the Eating Attitudes Test-26 (EAT-26) [48,49]. Family financial status was assessed through a single self-reported question, with participants categorizing their status as low, average, or high.

#### 2.3.1. Internet Addiction

The IAT scale contains 20 items in which participants were asked to rate how often they experience symptoms of harmful internet use, such as excessive time spent online, neglecting daily obligations, decline of academic or professional performance, concealing the time spent using the internet, and behaviors such as insufficient sleep, social isolation, depressed feelings, and unsuccessful attempts to limit internet use. Each item is scored from 0 to 5, with possible total scores ranging from 0 to 100. A score of 0 to 30 reflects a normal level of internet use, 31 to 49 points indicate the presence of a mild level of IA, and 50 to 79 reflect the presence of a moderate level of IA, while a score of 80 to 100 indicates severe internet addiction [44,45]. Due to the small number of participants in the moderate IA category and the absence of severe IA cases, mild and moderate IA categories were combined in subsequent analyses.

#### 2.3.2. Adherence to the Mediterranean Diet

KIDMED is a scale for assessing the degree of adherence of children and adolescents to the Mediterranean dietary pattern. It is a 16-item questionnaire including 12 items positively associated with the Mediterranean diet (scored + 1) and 4 items negatively associated with it (scored − 1), resulting in a score typically ranging from 0 to 12. The sum of the values is classified into one of the following categories: low adherence (≤3), medium adherence (4–7), and high adherence (≥8) [46,47].

#### 2.3.3. Disordered Eating Behavior

The EAT-26 is a widely used 26-item scale that measures eating attitudes and behaviors. It consists of the following three sub-scales: dieting (thirteen items), bulimia/dietary preoccupation (six items), and oral control (seven items). Total scores range from 0 to 78 and a total score ≥ 20 suggests a high risk for eating disorders and the need for further clinical evaluation [48,49].

#### 2.3.4. Anthropometric Data

Τhe Body Mass Index (BMI) for each participant was calculated according to the values of their self-reported height and weight, as body weight (kg) divided by height (m^2^). Young adults were classified as underweight (BMI < 18.5 kg/m^2^), normoweight (BMI 18.5–24.9 kg/m^2^), overweight (BMI 25.0–29.9 kg/m^2^) and obese (BMI ≥ 30 kg/m^2^). Participants younger than 18 years were classified according to the age- and sex-specific International Obesity Task Force (IOTF) BMI cut-offs, which correspond to adult BMI thresholds of 16, 17, 18.5, 25, and 30 kg/m^2^ at age 18 years.

### 2.4. Statistical Analysis

Descriptive statistics (means and standard deviations) and relative frequencies (percentages) were used for demographic data as well as IAT, EAT-26, and KIDMED questionnaire variables, as appropriate. Associations between the scores and socio-demographic characteristics were evaluated using Pearson’s chi-squared test (x^2^). For analyses involving education level, only high school and university students were included, as these represented the primary and mutually comparable groups. Participants who reported employment, exam preparation, or postgraduate/graduate status were excluded from education-level subgroup analyses. Consequently, the total N varies slightly across tests due to missing or inapplicable responses. A logistic regression analysis was conducted to evaluate the association between the risk of eating disorders (dependent variable) and various predictors, including gender, educational status, level of urbanization, financial status, internet addiction, and adherence to the Mediterranean diet. All predictors were first examined in univariate models; variables of primary interest and/or those showing evidence of association were then entered into a multivariate model. Odds ratios (ORs), 95% confidence intervals (CIs), and *p*-values were reported for each predictor, with categorical variables analyzed relative to reference groups. Statistical analysis was performed with the IBM Statistical Package for the Social Sciences (SPSS) v. 27.0 (IBM Corp., Armonk, NY, USA).

## 3. Results

### 3.1. Description of the Study Sample

Of the 212 participants (N = 212), 33.5% (n = 71) were males and 64.2% (n = 136) females, while five participants (2.4%) stated that they preferred not to answer. Of the 212 participants, 51.4% (n = 109) were high school students and 43.9% (n = 93) were university students. In addition, 3.3% (n = 7) reported having a job, 2.4% (n = 5) were in university exam preparation, 0.9% (n = 2) were graduates, and 0.5% (n = 1) were postgraduate students. Participants’ median age was 18 years (interquartile range IQR = 16–21.5) Regarding their place of residence, 16% (n = 34) of participants stated that they lived in a rural area and 27.8% (n = 59) in a semi-urban area, while the remaining 56.1% (n = 119) lived in urban areas. As regards the self-reported financial status of their family, 43.9% (n = 93) stated that it was high, 54.2% (n = 115) that it was average and 1.9% (n = 4) that it was low. With reference to BMI, 24 participants (11.3%) were underweight, 143 (67.5%) were of normal weight, 35 (16.5%) were overweight and 10 participants (4.7%) were obese.

### 3.2. Descriptive Analysis of IA According to Socio-Demographic Characteristics

This analysis was conducted to provide a descriptive overview of internet addiction across socio-demographic characteristics of the sample. Overall, 148 of the 212 participants (69.8%) presented normal levels of internet use, 49 participants (23.1%) presented mild IA, and 15 participants (7.1%) presented moderate IA; none of the participants in this study experienced severe IA. No significant associations were observed between IA and education level, gender, or financial status (all *p* > 0.05). A significant association was observed with urbanization (*p* = 0.014), with mild IA being more frequent in urban areas, while moderate IA was relatively more common in rural/semi-urban areas (Table 1). Due to the small number of participants in the moderate IA category and the absence of severe IA cases, mild and moderate IA categories were combined in subsequent analyses.

### 3.3. Correlates of Adherence to MD

Data on the dietary habits of the participants (N = 212) according to their answers to the KIDMED questionnaire, showed that 20 participants (9.4%) showed low MD adherence (0–3), 112 (52.8%) moderate adherence (4–7) and 80 participants (37.7%) high MD adherence (≥8). No significant associations were observed between MD adherence and education level, gender, financial status, or urbanization (all *p* > 0.05) (Table 2).

### 3.4. Correlates of Risk for Eating Disorders

In relation to the EAT-26 questionnaire, 33 (15.6%) of the 212 participants were at risk for EDs (score above 20), while the remaining 179 (84.4%) scored in the normal range (score 0–19). No significant associations were observed between risk of EDs and education level, financial status, or urbanization (all *p* > 0.05). A significantly higher proportion of females were at risk for EDs compared to males (21.3% vs. 2.8%, *p* < 0.001). No significant association was observed between IA and risk of EDs in the chi-square analysis (*p* = 0.210) (Table 3).

### 3.5. Univariate and Multivariate Analysis of Predictors of Eating Disorder Risk

To further examine these associations and estimate their magnitude, univariate (Table 4) and multivariate (Table 5) logistic regression analyses were performed. Univariate logistic regression analysis revealed significant associations between gender, internet addiction levels, and the risk of eating disorders. Females had significantly higher odds of being at risk for eating disorders compared to males (OR = 9.35, 95% CI: 2.16–40.44, *p* = 0.003). Moderate internet addiction was also significantly associated with higher odds of eating disorder risk compared to no internet addiction (OR = 7.31, 95% CI: 2.4–22.4, *p* < 0.001). However, mild internet addiction was not significantly associated with the outcome (OR = 0.73, 95% CI: 0.26–2.05, *p* = 0.55).

Other variables, including educational status, urbanization, financial status, and adherence to the Mediterranean diet (MD compliance), were not significantly associated with eating disorder risk. University students had lower odds of risk compared to high school students, though the association was not significant (OR = 0.56, 95% CI: 0.26–1.24, *p* = 0.153). Urban residents showed lower odds compared to rural/semi-urban residents (OR = 0.69, 95% CI: 0.33–1.46, *p* = 0.337), and individuals with high financial status had similar odds compared to those with medium/low financial status (OR = 0.93, 95% CI: 0.44–1.98, *p* = 0.856). Similarly, medium/high MD compliance was not significantly different from low compliance (OR = 1.05, 95% CI: 0.29–3.08, *p* = 0.942).

These associations remained significant in the multivariate analysis, with females showing markedly higher odds of eating disorder risk compared to males (OR = 9.28, 95% CI: 2.10–40.91, *p* = 0.003). Moderate internet addiction was strongly associated with increased risk of eating disorders compared to no internet addiction (OR = 6.70, 95% CI: 1.71–26.35, *p* = 0.006). Other factors, including educational status and Mediterranean diet compliance, were not significantly associated with eating disorder risk in the adjusted model.

## 4. Discussion

The pilot study was conducted in order to explore the relationship between IA, EDs and the participants’ eating habits according to MD compliance as well as the correlation of socio-demographic characteristics in a total sample of 212 Greek students aged 15–18 years and university students aged 18–24 years (N = 212). Regarding IA, our research findings revealed that a total of 30.2% experienced IA; more specifically 49 participants showed mild IA (23.1%) and 15 moderate IA (7.1%), while none of the participants showed severe IA. Among the high-school adolescents and university students 28.4% and 34.4% presented IA, respectively. The percentages of IA in our research are higher than the ones presented in the research of Diakos and Doukas, 2010 [25,50], in which the IA rate was 11.82%. In a recent survey of the Hellenic Center for Safe Internet in 2019 (n.d.) approximately 20% students either admit or think they might have a problem with IA [51]. Compared with findings from 2010 and 2019, the present study reported a higher prevalence of internet addiction, with the 2019 survey also showing higher rates than the 2010 study, indicating a potential upward trend over time among school students. This pattern may be influenced by earlier and more pervasive internet use, greater connectivity, and the widespread use of smartphones.

As far as the university students are concerned, the rates of the present research could also be characterized as somewhat increasing in relation to those noted by Tsouvelas and Giotakou (2011) in which 70.2% of participating students make normal use of the internet, 26% are at risk for pathological engagement with it and 3.5% show symptoms of pathological preoccupation [52]. Higher rates were found by a more recent survey by Kakouris and Tsimouris (2021) involving a sample of 100 students which identified 49% with mild levels of IA and 11% with moderate IA [53]. These upward trends in the recent years may be linked to the widespread use of social media in recent years, as well as the heightened social isolation and increased reliance on digital communication during the COVID-19 pandemic.

Compared to the international literature, the prevalence of IA in our study appears higher than pooled estimates reported in a meta-analysis by Pan et al. (2020) [54], while individual studies, such as Seki et al. (2019), highlight variability across populations [55].

Previous research has reported higher rates of IA among male students [56]. In our sample, however, the pattern was not as clear. Although the proportion of mild IA was slightly higher in males than in females (25.4% vs. 22.1%), moderate IA was more common in females (7.4% vs. 2.8%). Importantly, these differences were not statistically significant (*p* = 0.390), indicating that male and female participants in this study exhibited similar overall levels of internet use. This may reflect a narrowing gender gap in internet behaviors, possibly due to widespread access to digital devices and similar patterns of online engagement across genders.

Regarding the participants’ eating habits and MD compliance, 9.4% of the participants (n = 20) in this study, showed eating habits of low MD compliance, 52.8% (n = 112) moderate MD compliance and 37.7% (n = 80) high MD compliance. The findings of a panhellenic survey on the behavior related to adolescent health [57], which states that the diet of adolescents seems to improve between the years 2006 and 2014, may explain the results of the present research, where the percentage of participants with high MD compliance standards can be described as encouraging. Recent research data from the Transnational Report of the Health Behavior in School-aged Children (HBSC)/World Health Organization (WHO) Research Program (2018), however, show that adolescents in Greece are at a higher percentage overweight or obese (25%) than the results of the HBSC program overall (21%) [58]. Although HBSC data refer to younger adolescents (11–15 years), and therefore are not directly comparable to our sample, they provide useful contextual information on broader trends in adolescent health in Greece. The results of our survey, which show that 21.2% of high school and university students are overweight and obese, seem to converge more closely with the results of the HBSC/WHO survey (2018) as well as the results of Androutsos et al. (2021) who report that although junk food consumption has decreased, body weight increased in 35% of children and adolescents during the restrictive measures against COVID-19 [59].

The results of the present research should also be evaluated in relation to the meta-analysis of García Cabrera et al. (2015) on MD compliance, using the KIDMED questionnaire, which also includes Greek surveys [14]. The pooled analysis shows that the percentage of participants with high MD was 10%, while the percentage of those with low MD compliance amounted to 21%. The findings in the analysis by country group is worth mentioning since the rate of low MD compliance is 28% for Greece, Cyprus and Turkey, and 11% for Spain and Chile [14]. In addition, the results of the research by Mazaraki et al. (2011), which was used in the meta-analysis of García Cabrera et al. (2015), show a large deviation from the findings of the present research as 42% of adolescents showed low, 51.2% moderate and only 6.8% high MD compliance on the KIDMED [14,60]. Similar were the results of the research by Lazarou et al. (2009), which was also used in the same meta-analysis, with a sample of 1140 children in Cyprus, and which found that only 6.7% of the sample was classified as having high MD compliance, while 37% had a low score in the KIDMED, which contradicts the findings of the present study [14,61].

With regard to the relationship between IA and disordered eating, 15.6% (n = 33) of the participants in the present study scored above the EAT-26 cut-off, indicating an elevated risk for an eating disorder. The analysis demonstrated that the risk of disordered eating was significantly associated with female sex and moderate IA, while mild IA, degree of urbanization, MD compliance, and financial status showed no significant associations. The lack of association between Mediterranean diet adherence and both internet addiction and disordered eating behaviors is noteworthy. One possible explanation is that adherence to the Mediterranean diet reflects overall dietary quality rather than specific eating attitudes or pathological behaviors, which are more directly captured by instruments such as the EAT-26. Additionally, disordered eating behaviors are complex and influenced by psychological, social, and environmental factors that may not be fully reflected in dietary patterns alone. It is also possible that the KIDMED index, while widely used, may not be sufficiently sensitive to detect subtle differences in eating behaviors related to disordered patterns in this age group. In the multivariate model, female sex and moderate IA remained significant predictors of increased ED risk, whereas being a university student—as compared to a high school student—was marginally non-significant. The fact that only moderate, but not mild, IA was associated with increased risk of disordered eating behaviors may suggest a threshold effect, whereby more severe and persistent patterns of internet use are required to influence eating-related attitudes and behaviors. Moderate IA may reflect greater exposure to potentially harmful online content, such as appearance-focused social media, as well as more pronounced disruptions in daily routines, including sleep and eating patterns, which could contribute to disordered eating. In contrast, mild IA may represent less intensive or less disruptive use, insufficient to produce measurable behavioral effects. These results suggest that both gender-related vulnerabilities and higher levels of problematic internet use may play a meaningful role in the development of disordered eating behaviors, highlighting the need for targeted prevention and early identification strategies. Additionally, higher levels of internet use may be associated with sedentary lifestyles and irregular eating patterns, which could further influence eating behaviors. Gender differences may also reflect greater societal pressure on females regarding body image and weight, making them more vulnerable to such influences.

Since there are no corresponding Greek studies examining the combined relationship between IA, ED risk, and MD compliance, comparisons can only be made with studies conducted in other countries. First, research carried out by Alpaslan et al. (2015) in a sample of high school students in Turkey presented results similar to ours with the percentage of participants experiencing EDs being 15.2%, possibly indicating a similar prevalence of EDs in the two neighboring countries [38]. In contrast to the findings of Alpaslan et al. (2015), who reported that IA was associated with gender, BMI, and eating disorder symptoms, our study did not identify significant associations between IA and gender, educational status, or financial status [38]. IA in our sample was significantly associated only with urbanization. Because BMI, MD compliance, and eating disorder symptoms were not examined as predictors of IA, future studies should explore whether these factors also contribute to problematic internet use in this population. In addition, in the survey of Canan et al. (2014) among adolescent students in Turkey it was reported that 3.8% with severe IA and 5.3% with mild IA were at increased risk of developing EDs [41].

These findings are consistent with previous research across different populations, which has demonstrated a positive association between problematic internet use and disordered eating behaviors, often with higher IA prevalence in males and greater ED risk in females [21,33,34]. Additional studies suggest that increased engagement with social media and body image concerns may contribute to eating pathology, particularly among female adolescents and young adults [62,63]. Evidence from studies in Asian populations further supports the association between IA and disordered eating behaviors, including binge eating and weight-related concerns [35,42]. Finally, a meta-analysis by Hinojo-Lucena et al. (2019) confirmed that problematic internet use is a significant predictor of eating disorders in student populations [22].

### Strengths and Limitations of the Study

The strongest point of the study lies in the fact that it is the first study in Greece that explored the correlation between ED and IA and compliance with the MD and the quality of nutrition of adolescents and young adults—students. The present study, therefore, can be a starting point for more focused research with a larger and/or clinical sample either of the same age groups or of younger or older age. Another strong point of the study that should be mentioned is that three questionnaires validated for the Greek population were used in combination with the collection of demographic and anthropometric data. It is also worth noting that the sample of participants came from various regions of our country, which makes it partially representative. Finally, the number of participants can be characterized as adequate for a pilot study.

The present cross-sectional study presents some limitations. The first limitation refers to the cross-sectional nature of the study which does not allow establishing a causal relationship between the study variables. Also, the small sample size does not allow generalization of the results to the wider population of high school and university students. Furthermore, no participants in the present study were classified as having severe IA, which may have limited the ability to examine associations across the full spectrum of IA severity and potentially underestimated the strength of relationships involving more severe forms of internet addiction. An additional limitation of the study is the gender imbalance of the sample, with females being overrepresented. This may be attributed to the voluntary, self-selected nature of participation in the online survey. As a result, the findings may not be fully generalizable to male students, and potential gender-related differences should be interpreted with caution. The method of data collection also places limitations on the generalizability of the research results, as participation was voluntary. It is possible that individuals with more concerning behaviors (e.g., higher levels of internet addiction or disordered eating) were less likely to participate or complete the survey. Consequently, self-selection bias may have occurred, potentially leading to an underestimation of these behaviors in the study sample. It is equally possible that there were subjects who, while they started filling in the questionnaire, stopped it at some stage, perhaps because they felt uncomfortable with the questions they had to answer. It should also be noted that since the data collection is from a self-report questionnaire, conclusions should be drawn with caution. In addition, family financial status was assessed using a single self-reported item rather than a validated socioeconomic measure, which may limit the precision of this variable. Additionally, parental educational level was not assessed, although it is an important socioeconomic indicator that may influence dietary habits and behavioral outcomes. Moreover, anthropometric data (height and weight) were self-reported, which may introduce reporting bias, as participants may overestimate height and underestimate weight, potentially leading to an underestimation of BMI. This may partly explain the relatively low proportion of overweight and obese participants observed in the sample. Also, the inclusion of both high-school and university students in the same analyses may have introduced heterogeneity, as these groups differ in developmental stage and lifestyle characteristics; therefore, the findings should be interpreted with caution. Nevertheless, the use of an anonymous online self-report questionnaire can be a facilitating factor for honest disclosure of sensitive or disturbing behavior. Finally, other relevant lifestyle factors, such as smoking and physical activity, were not assessed, which may have limited the ability to account for potential confounding variables.

## 5. Conclusions

The present study on the investigation of the relationship between EDs, compliance with the MD, and IA constitutes a first step for a possible future more expanded and targeted study of this specific topic in Greece. The findings are generally consistent with previous research showing increased ED risk among females and a link between higher levels of internet addiction and disordered eating behaviors. In our study, although there were no individuals scoring extremely high on the EAT-26, probably due to the relatively small sample, the fact that approximately one sixth of the sample was at risk for EDs can be considered worrying, as there may be a potential risk for its further increase if initiatives are not adopted to address it. This risk, as shown by the present study and the literature, is greater for females, which makes the need for interventions in this population group more urgent, while the fact that no statistically significant difference was found between the subgroups of schoolchildren and university students can be attributed to the fact that these ages present quite common characteristics in the way they communicate and entertain themselves.

Finally, regarding the participants’ compliance with the MD and the quality of their dietary habits, the results of the present study are relatively encouraging, since 37.7% present excellent compliance with the MD. However, the fact that approximately one tenth of the participants have low compliance is a cause for concern and should create awareness for immediate interventions.

As already mentioned, the results of this research are based on self-reports of the participants. Targeted qualitative and clinical research could investigate the results more specifically and clinically evidenced, extending the study to a population with IA and/or EDs in the future. It would also be interesting to study the cognitive and psychological factors associated with IA and DEB, in order to have a more spherical exploration of the issue. Finally, a scale that would investigate the relationship of IA with EDs could be created and calibrated as a useful tool for researchers in the specific field.

Future research should include larger and more representative samples, as well as longitudinal designs, in order to better understand causal relationships between internet addiction, dietary behaviors, and disordered eating. In addition, interventions targeting problematic internet use and promoting healthy eating behaviors—particularly among female adolescents and young adults—may be beneficial. Integrating digital literacy and mental health support into prevention programs could also contribute to reducing the risk of disordered eating behaviors.

## Figures and Tables

**Table 1:** Correlates of IA in the sample.

Internet Addiction Level	Group 1 n (%)	Group 2 n (%)	χ^2^	*p*-Value
Current level of education	High school students	University students		
Normal use	78 (75.0)	61 (65.6)		
Mild IA	24 (22.0)	24 (25.8)	0.88	0.643
Moderate IA	7 (6.4)	8 (8.6)		
Gender	Male	Female		
Normal use	51 (71.8)	96 (70.6)		
Mild IA	18 (25.4)	30 (22.1)	1.88	0.390
Moderate IA	2 (2.8)	10 (7.4)		
Financial status	Medium/Low	High		
Normal use	79 (66.4)	69 (74.2)		
Mild IA	28 (23.5)	21 (22.6)	3.95	0.139
Moderate IA	12 (10.1)	3 (3.2)		
Urbanization	Rural/Semi-urban	Urban		
Normal use	71 (76.3)	77 (64.7)		
Mild IA	13 (14.0)	36 (30.3)	8.58	0.014
Moderate IA	9 (9.7)	6 (5.0)		

Note: Valid N varies across analyses due to missing or inapplicable responses: education level (N = 202), gender (N = 207), financial status (N = 212), urbanization (N = 212). IA: Internet Addiction.

**Table 2:** MD adherence in the sample; evaluation of associations with sociodemographic variables.

MD Adherence	Group 1 n (%)	Group 2 n (%)	χ^2^	*p*-Value
Current level of education	High school students	University students		
Low (≤3)	10 (9.2)	10 (10.8)		
Medium (4–7)	55 (50.5)	53 (57.0)	1.43	0.490
High (≥8)	44 (40.4)	30 (32.3)		
Gender	Male	Female		
Low (≤3)	9 (12.7)	11 (8.1)		
Medium (4–7)	42 (59.2)	68 (50.0)	4.12	0.127
High (≥8)	20 (28.2)	57 (41.9)		
Financial status	Medium/Low	High		
Low (≤3)	15 (12.6)	5 (5.4)		
Medium (4–7)	64 (53.8)	48 (51.6)	4.16	0.125
High (≥8)	40 (33.6)	40 (43.0)		
Urbanization	Rural/Semi-urban	Urban		
Low (≤3)	8 (8.6)	12 (10.1)		
Medium (4–7)	46 (49.5)	66 (55.5)	1.252	0.535
High (≥8)	39 (41.9)	41 (34.5)		

Note: Valid N varies across analyses due to missing or inapplicable responses: education level (N = 202), gender (N = 207), financial status (N = 212), urbanization (N = 212). MD: Mediterranean Diet.

**Table 3:** Correlates of risk for eating disorders (EDs) in the sample.

Risk for EDs (EAT-26 ≥ 20)	Group 1 n (%)	Group 2 n (%)	χ^2^	*p*-Value
Current level of education	High school students	University students		
Risk for EDs	21 (19.3)	11 (11.8)	2.08	0.149
Normal eating habits	88 (80.7)	82 (88.2)		
Gender	Male	Female		
Risk for EDs	2 (2.8)	29 (21.3)	12.55	<0.001
Normal eating habits	69 (97.2)	107 (78.7)		
Financial status	Medium/Low	High		
Risk for EDs	19 (16.0)	14 (15.0)	0.03	0.856
Normal eating habits	100 (84.0)	79 (85.0)		
Urbanization	Rural/Semi-urban	Urban		
Risk for EDs	17 (18.3)	16 (13.45)	0.928	0.335
Normal eating habits	76 (81.72)	103 (86.55)		
Internet Addiction Level	Normal	Mild IA/Moderate IA		
Risk for EDs	20 (13.5)	13 (20.3)	1.571	0.210
Normal eating habits	128 (86.5)	51 (79.7)		

Note: Valid N varies across analyses due to missing or inapplicable responses: education level (N = 202), gender (N = 207), financial status (N = 212), urbanization (N = 212), internet addiction level (N = 212). ED: Eating Disorder; IA: Internet Addiction.

**Table 4:** Univariate logistic regression analysis with risk of eating disorders (EDs) as the dependent variable.

	OR	95% CI	*p*-Value
Gender			
Male	Ref.		
Female	9.35	2.16–40.44	0.003
Educational Status			
High school student	Ref.		
University student	0.56	0.26–1.24	0.153
Urbanization			
Rural/Semi-urban	Ref.		
Urban	0.69	0.33–1.46	0.337
Financial status			
Medium/low	Ref.		
High	0.93	0.44–1.98	0.856
Internet Addiction			
No	Ref.		
Mild	0.73	0.26–2.05	0.55
Moderate	7.31	2.40–22.40	<0.001
MD compliance			
Low (0–3)	Ref.		
Medium/High (≥4)	1.05	0.29–3.08	0.942

Note: ED: Eating Disorder; MD: Mediterranean Diet; OR: Odds Ratio; CI: Confidence Interval. Reference categories are indicated as “Ref.” Dependent variable: risk of eating disorders (EDs), based on EAT-26 score ≥ 20. Valid N varies slightly across models due to missing data.

**Table 5:** Multivariate logistic regression analysis with risk of eating disorders (EDs) as the dependent variable.

	OR	95% CI	*p*-Value
Internet Addiction			
No	Ref.		
Mild	0.86	0.29–2.55	0.783
Moderate	6.70	1.71–26.35	0.006
Gender			
Male	Ref.		
Female	9.28	2.10–40.91	0.003
Educational Status			
High school student	Ref.		
University student	0.45	0.19–1.09	0.078
MD Compliance			
Low (0–3)	Ref.		
Medium/High (≥4)	0.83	0.20–3.56	0.806

Note: ED: Eating Disorder; MD: Mediterranean Diet; OR: Odds Ratio; CI: Confidence Interval. Reference categories are indicated as “Ref.” Dependent variable: risk of eating disorders (EDs), based on EAT-26 score ≥ 20. Valid N reflects complete-case analysis.

## Data Availability

The original data presented in this study are available on request from the corresponding author.

## Abbreviations

The following abbreviations are used in this manuscript:

IA	Internet Addiction
DEB	Disordered Eating Behavior
MD	Mediterranean Diet
IAT	Internet Addiction Test
EAT-26	Eating Attitudes Test
OR	Odds Ratio
ED	Eating Disorders
AN	Anorexia Nervosa
BN	Bulimia Nervosa
BED	Binge Eating Disorder
BMI	Body Mass Index
IOTF	International Obesity Task Force
CI	Confidence Interval
SPSS	Statistical Package for Social Sciences
HBSC	Health Behavior in School-aged Children
WHO	World Health Organization
